# Transient hypoglossal nerve palsy after transoral intubation for general anesthesia

**DOI:** 10.12669/pjms.333.11662

**Published:** 2017

**Authors:** Young Jin Choi, Jin Hee Lee, Young Duck Shin

**Affiliations:** 1 Young Jin Choi, MD, PhD. Department of Surgery, College of Medicine, Chungbuk National University, Korea; 2 Jin Hee Lee, MD. Department of Anesthesiology and Pain Medicine, College of Medicine, Chungbuk National University, Korea; 3 Young Duck Shin, MD, PhD. Department of Anesthesiology and Pain Medicine, College of Medicine, Chungbuk National University, Korea

**Keywords:** Complication, Hypoglossal nerve, Intubation

## Abstract

Hypoglossal nerve palsy induces palsy in the ipsilateral lingual muscles, resulting in tongue deviation and articulation disorder. It is a rare condition that may stem from a variety of causes. Therefore, it is important to consider the possible causes of hypoglossal nerve palsy related to surgery or anesthesia, including intubation, the surgical positions, and mask ventilation during recovery.

## INTRODUCTION

The hypoglossal nerve is the twelfth cranial nerve, and is a pure motor nerve that is involved in the movements of the tongue and infrahyoid muscles. Hypoglossal nerve palsy induces palsy in the ipsilateral lingual muscles, causing tongue deviation and articulation disorder. It is a rare condition that may stem from a variety of causes.

The most common cause is a tumor in the innervation path of the hypoglossal nerve, followed by trauma. Other causes include cerebral infarction, deformity, multiple sclerosis, dehydration polyneuropathy, infection, and diabetes.^1^ Cases of isolated hypoglossal nerve palsy without any accompanying neurological symptoms are very rare, and are classified as idiopathic if a clear cause is not found. The laryngoscope inflicted direct pressure on the hypoglossal nerve during transoral intubation is another possibility. The authors of this study report a rare case of contralateral hypoglossal nerve palsy after general anesthesia, together with the possible causes and precautions.

## CASE PRESENTATION

A 53-year-old male patient (75kg, 172cm) received general anesthesia (GA) for retinal detachment surgery. The patient had no notable medical history other than diabetes, and his preoperative test findings were all within the normal range. GA was induced with propofol 120mg, vecuronium 8mg, and glycopyrolate 0.2mg and the patient was mask-ventilated for 3 minutes with 100% oxygen and 7.0 Vol% Sevoflurane. There were no particular difficulties in maintaining airway via a mask. After additionally administering 1 mg of alfentanyl, the endotracheal intubation was performed with a Macintosh size 3 laryngoscope blade. The Cormack-Lehane grade 1 view was obtained and patient was intubated using 7.5-mm-ID endotracheal tube at the first attempt. The tube was fixed at about 23 cm to right lower lip.

The anesthesia was maintained with 1.5 L/min of oxygen, 1.5 L/min of nitrogen, sevoflurane MAC 1.0-1.2, and there were no noticeable changes in the patient’s vital signs during the surgery. After the surgery was completed, the patient was extubated upon confirmation of recovery of spontaneous breathing (TOF ratio >0.9). However, as the airway was obstructed immediately after the extubation, we inserted an oral airway by fixing the mask with triple airway maneuver. Soon after, the obstruction disappeared naturally, and breathing was recovered.

It took less than 2 minutes for natural recovery, and the SpO2 was maintained at 100%. The patient did not show any abnormal symptoms and was moved to the recovery room. The total length of anesthesia was 120 minutes.

**Fig.1:**
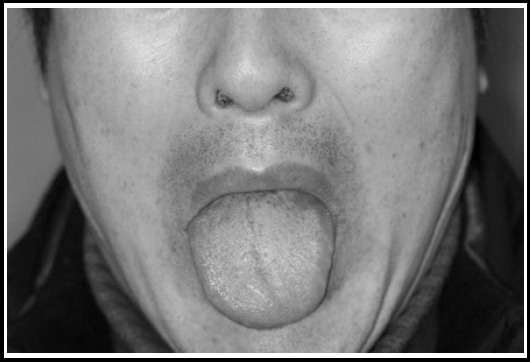
Photograph showing the tongue deviation to the left side when the patient was asked to protrude his tongue.

In the recovery room, the patient only had minor pain in the surgical area. The following morning, he showed dysarthria and complained of difficulty in moving his tongue. When he showed his tongue, a leftward deviation was clearly observed (Fig.1). However, there were no signs of intrinsic muscle atrophy or fasciculation. Moreover, there were no other symptoms of cranial nerve palsy, such as ageusia, facial palsy, reduced facial sensation, loss of gag reflex, and uvular deviation. We referred the patient to the neurology department under a suspicion of left hypoglossal nerve palsy. Although we did not perform facial CT, the neurology department recommended outpatient follow-up if no additional neurological symptoms were present, and the patient was discharged two days after the surgery. At the 1 week postoperative follow-up, the patient pointed out his slurred speech has slightly improved, although the leftward deviation of the tongue was still present. At the 4 weeks follow-up, the patient showed complete recovery.

## DISCUSSION

In hypoglossal nerve palsy related to surgery or anesthesia, the possible causes—including intubation, the surgical positions, and mask ventilation during recovery—must be considered.^2^

In our opinion, there may be some possible causes of hypoglossal nerve palsy in this case report. First, the pathologies related to intubation may involve the laryngoscope inflicting direct pressure on the hypoglossal nerve —which runs superficially— while moving the tongue forward, or inducing tension in the hypoglossal nerve when moving the head back for intubation.^3^ Second, compression on the cricoid cartilage during intubation will anatomically fix the superficially distributed hypoglossal nerve at the mandible angle, and if the intubation is performed in this condition, the nerve may be hyperextended.^4^

Third, fixing the tube in an abnormal location, excessive cuff pressure, the inflation of the cuff in the larynx rather than in the trachea, or extubating while the cuff is inflated may cause hypoglossal palsy.^5^

As there was no problem with the position of the head during the surgery, the hypoglossal nerve palsy may have been caused by the traction during intubation. There is also a possibility that the intubated tube compressed the hypoglossal nerve, or that damage was inflicted to the nerve by direct traction on the mandible angle. However, considering the fact that the tube was fixed in the normal position and that the operation was relatively short, it seems unlikely that the hypoglossal nerve palsy was caused by either the endotracheal tube or by excessive pressure in the cuff. However, as the palsy symptoms were transient and the patient fully recovered, it is presumed that it was a transient impairment that was caused by nerve tension from typical pressure and traction.

The prognosis of hypoglossal nerve palsy varies widely, from permanent paralysis to complete recovery. Dziewas and Ludemann studied the prognosis of hypoglossal nerve palsy that occurred after procedures related to the oropharynx region—such as endotracheal intubation, larynx mask airway, and bronchoscopy—in 20 cases. They found that the patients had shown complete recovery within 4 months in 65%, partial recovery in 15%, and no recovery in 20%.^6^

The treatment of hypoglossal nerve palsy should be selected according to the lesion, the damaged nerve, and the underlying disease. Although there has been a report of permanent nerve damage, patients with transient hypoglossal palsies are known to fully recover within 6 months of incidence.

Postoperative hypoglossal nerve palsy may cause great discomfort as it is related to speech, a critical aspect in daily life. Prophylactic measures include the avoidance of excessive traction of the laryngoscope during intubation and of hyperextension of the head and neck. In addition, anesthesiologists must take precautions against inflated cuffs when surgeries are prolonged.
